# Binary Surfactant–Mediated Tunable Nanotip Growth on Gold Nanoparticles and Applications in Photothermal Catalysis

**DOI:** 10.3389/fchem.2021.699548

**Published:** 2021-07-07

**Authors:** Xiaohu Mi, Tingting Zhang, Baobao Zhang, Min Ji, Bowen Kang, Chao Kang, Zhengkun Fu, Zhenglong Zhang, Hairong Zheng

**Affiliations:** School of Physics and Information Technology, Shaanxi Normal University, Xi’an, China

**Keywords:** nanotip growth, binary surfactants, surface plasmon resonance, photothermal effect, SERS

## Abstract

Plasmonic nanostructures with sharp tips are widely used for optical signal enhancement because of their strong light-confining abilities. These structures have a wide range of potential applications, for example, in sensing, bioimaging, and surface-enhanced Raman scattering. Au nanoparticles, which are important plasmonic materials with high photothermal conversion efficiencies in the visible to near-infrared region, have contributed greatly to the development of photothermal catalysis. However, the existing methods for synthesizing nanostructures with tips need the assistance of poly(vinylpyrrolidone), thiols, or biomolecules. This greatly hinders signal detection because of stubborn residues. Here, we propose an efficient binary surfactant–mediated method for controlling nanotip growth on Au nanoparticle surfaces. This avoids the effects of surfactants and can be used with other Au nanostructures. The Au architecture tip growth process can be controlled well by adjusting the ratio of hexadecyltrimethylammonium bromide to hexadecyltrimethylammonium chloride. This is due to the different levels of attraction between Br^−^/Cl^−^ and Au^3+^ ions. The surface-enhanced Raman scattering and catalytic abilities of the synthesized nanoparticles with tips were evaluated by electromagnetic simulation and photothermal catalysis experiments (with 4-nitrothiophenol). The results show good potential for use in surface-enhanced Raman scattering applications. This method provides a new strategy for designing plasmonic photothermal nanostructures for chemical and biological applications.

## Introduction

Plasmonic nanoparticles (NPs) are promising materials for nanoscale light confinement and manipulation, and have been widely used in identification (Liu et al., 2017; Qin et al., 2017; Ma et al., 2020; Mi et al., 2021), bioimaging (Mi et al., 2019; Wu et al., 2019; Falahati et al., 2020; Barella et al., 2021), and catalysis (Zhang et al., 2016; Zhang et al., 2019; Gelle et al., 2020). Because of their stable chemical properties, strong absorption, and wide-range tunable surface plasmon resonance (SPR) in the visible to near-infrared range (Huang et al., 2014; Ruan et al., 2014; Li et al., 2015; Sanchez-Iglesias et al., 2017; Wang et al., 2018), Au NPs show strong photothermal and electromagnetic (EM) field enhancements. These have important roles in optical signal enhancement applications (Wei and Xu, 2013; Ding et al., 2019; Langer et al., 2020; Yin et al., 2020). Plasmonic structures with sharp edges or tips can enhance an EM field near the edge or tip area 1000-fold because of charge aggregation, and they can minimize the field confinement to several nanometers (Schröder et al., 2015; Bombail et al., 2019). This strong field enhancement by structures with nanotips provides a new approach to the precision control and detection of optical signals, and can improve the efficiency of surface-enhanced Raman scattering (SERS) (Zhang et al., 2019). The strong light-absorbing ability of Au NPs not only gives strong EM field enhancement but also has great potential for improving photothermal conversion. This has already been widely used in photocatalysis (Shi et al., 2019) (Zhou et al., 2016; Zhang et al., 2019; Kong et al., 2020; Kong et al., 2021; Mateo et al., 2021).

In the traditional method for synthesizing plasmonic photothermal nanostructures with nanotips, poly(vinylpyrrolidone), thiols, and biomolecules are used as surfactants (Barbosa et al., 2010; Liu et al., 2014; Fan et al., 2018; Shi et al., 2020). However, these surfactants are hard to remove and can greatly affect the veracity of the measured optical signal, especially in Raman signal detection (Ansar et al., 2013; Zhang et al., 2020a; Zhang et al., 2020b; Qu et al., 2021). In addition, previous methods only enable the synthesis of specific structures with nanotips, for example, nanospheres, and the basic structure cannot be arbitrarily changed. It is therefore important to develop simpler methods for synthesizing nanostructures with controllable nanotips, which avoid the effects of surfactants.

In this study, we developed an efficient route for nanotip growth on Au nanostructures at room temperature. Importantly, we also solved the problem of surfactant removal by using a binary surfactant, that is, hexadecyltrimethylammonium chloride/hexadecyltrimethylammonium bromide (CTAC/CTAB) instead of poly(vinylpyrrolidone), thiols, or biomolecules. Various Au nanoarchitectures were selectively synthesized by adjusting the CTAC/CTAB concentrations. Our synthetic method is simpler than the commonly used seed-mediated growth method. Furthermore, the Au nanoarchitecture nanotips give better EM and photothermal field enhancements because the light confinement is stronger. This method provides a new strategy for designing plasmonic nanostructures.

## Experimental

Chloroauric acid (HAuCl_4_·4H_2_O), CTAB, CTAC, NaBH_4_, AgNO_3_, and 4-nitrothiophenol (4-NTP, 80%) were purchased from Sigma-Aldrich. HCl, sodium citrate, ascorbic acid, H_2_SO_4_ (98%), and H_2_O_2_ (50%) were purchased from the Sinopharm Chemical Reagent Co., Ltd. (China). Deionized water was used in all experiments.

Au NPs were synthesized by using a wet-chemical method that has been described in detail in our previous publications (Han et al., 2016). HAuCl_4_·4H_2_O (0.01 M, 2 ml) was dissolved in boiled deionized water (78 ml) under stirring. Sodium citrate (0.1 M, 8 ml) was quickly added to the solution. The mixture was kept at 100°C for 15 min under continuous stirring. The synthesized NPs were washed and centrifuged several times and then dispersed in water. They were stored in the dark at room temperature.

In a typical synthesis of other Au nanoarchitectures, HAuCl_4_ (0.01 M, 1 ml) aqueous solution was added to a CTAC/CTAB (0.1 M, 20 ml) aqueous solution, and the mixture was vigorously stirred for 2 min to obtain a homogeneous solution. Then ascorbic acid (0.1 M, 0.16 ml) aqueous solution, HCl (0.2 M, 0.2 ml), and Au NPs (200 µM) were added, and the mixture was stirred vigorously for 2 min. The mixture was then left undisturbed at room temperature for 2 h. The suspension was centrifuged at 3,000 rpm for 5 min, and the precipitate was collected, washed three times with ultrapure deionized water, and redispersed in deionized water (2 ml).

H_2_SO_4_/H_2_O_2_ solution was prepared by mixing H_2_SO_4_ (12 ml) and H_2_O_2_ (28 ml). A silicon substrate was immersed in the mixed H_2_SO_4_/H_2_O_2_ solution at 80°C for 2 h, and then washed with ethanol and deionized water. The Au nanoarchitectural species (200 µL) were added to 4-NTP (10^−7^ M, 4 ml). The silicon substrate was placed in the solution at 30°C for 12 h. The substrate was then washed with ethanol for 1 min and dried with highly pure N_2_ gas.

The samples were characterized by scanning electron microscopy (SEM) and transmission electron microscopy (TEM). The SEM images were obtained with an FEI-Nova Nano SEM 450 instrument at an operating voltage of 10 kV. The TEM images were obtained with a JEOL 2100 instrument at an accelerating voltage of 200 kV. Extinction spectra were recorded with a Perkin Elmer Lambda 950 spectrometer. All Raman and SERS spectra were obtained by using a Horiba Jobin Yvon LabRAM HR-Evolution Raman microscope with a ×100 objective (Olympus, NA = 0.9) and 1800 grooves mm^−1^ grating. The excitation wavelength was 633 nm. Each Raman spectrum was recorded with an accumulation time of 5 s.

**FIGURE 1:**
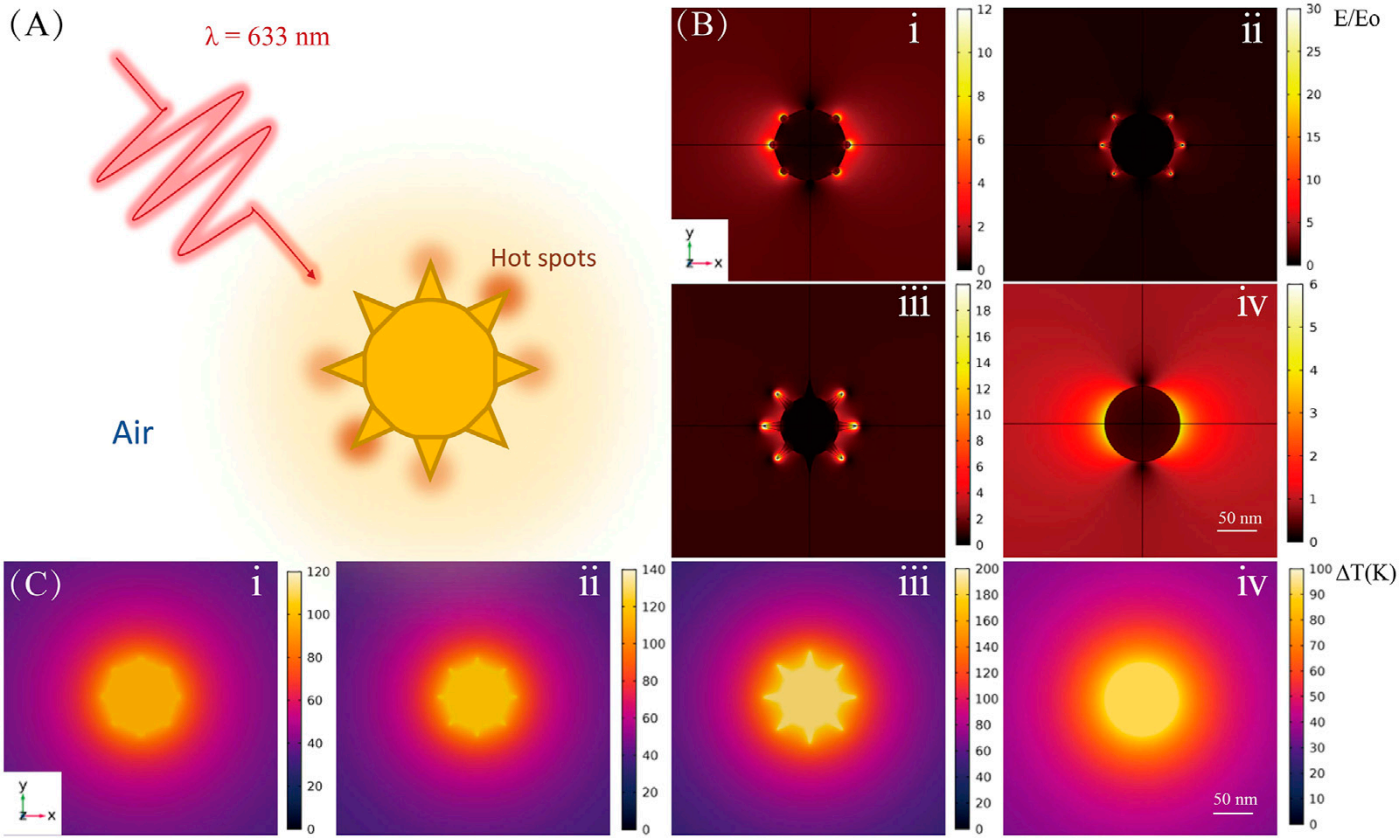
Theoretical calculations for Au NP tips of different shapes. **(A)** Schematic diagram of laser-excited Au NP. **(B)** Electromagnetic field and **(C)** temperature distributions for Au nanoarchitectures; i, ii, iii, and iv correspond to the images in Figure 2Dii, iv, v, and viii, respectively.

The finite element method (FEM) was used to perform numerical simulations of the EM field distribution. The electric and temperature field distributions were calculated by using the FEM; COMSOL software was used. The 633 nm incident light was shone from the *z*-direction and polarized in the *x*-direction. The average mesh size was set at 12 nm. The radius and tip size of the Au nanostructures were set in accordance with the experimental results shown in Figure 1.

## Results and Discussion

**FIGURE 2:**
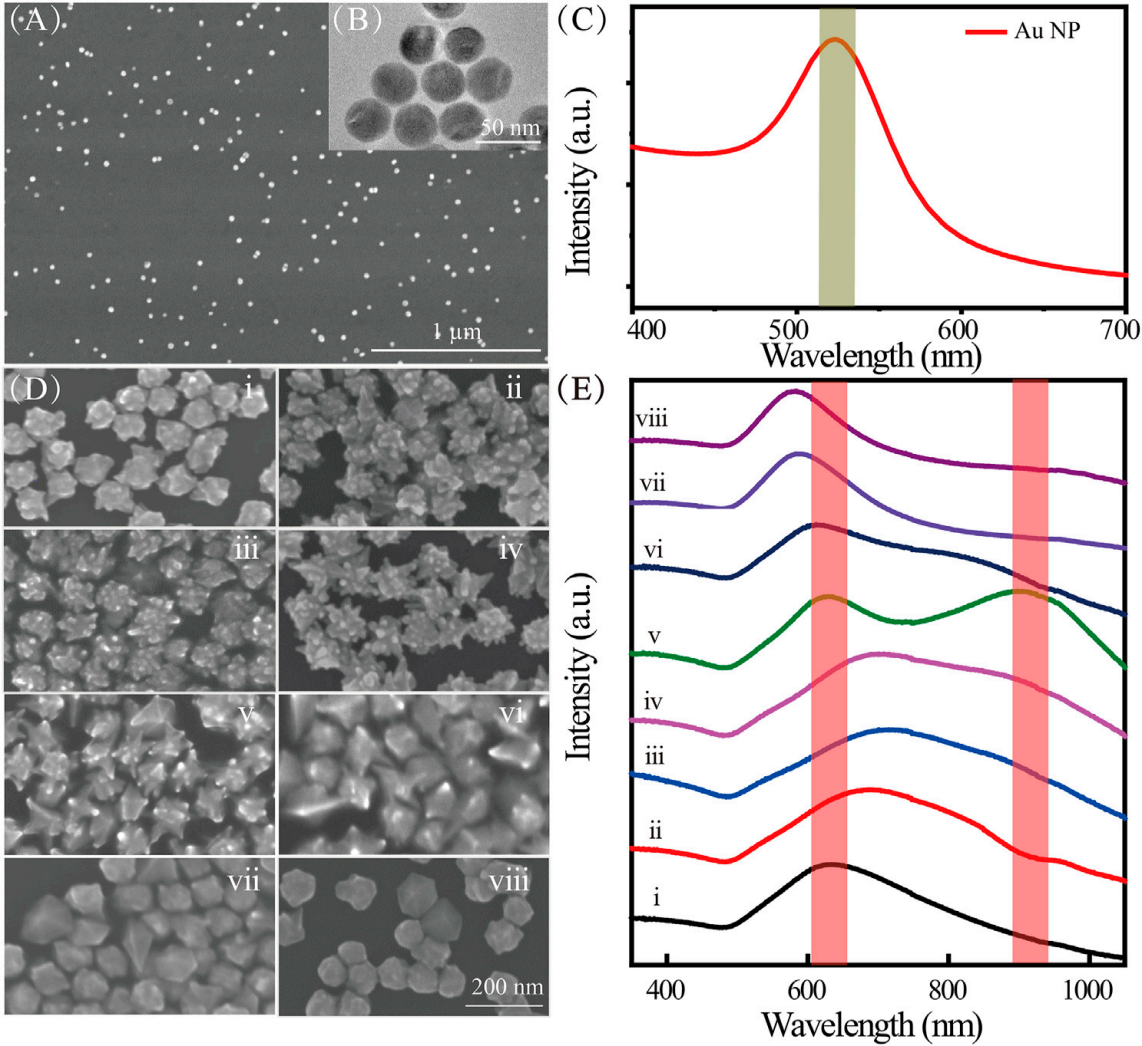
**(A)** SEM and **(B)** TEM images of Au NPs; **(C)** extinction spectra of Au NPs; **(D)** SEM images of Au nanoarchitectures synthesized with different concentrations of CTAC and CTAB; **(E)** extinction spectra of Au nanoarchitectures synthesized with different concentrations of CTAC and CTAB. i–viii correspond to different concentrations (mM) of CTAC and CTAB, namely, 100/0, 98.75/1.25, 96.9/3.1, 87.5/12.5, 62.5/37.5, 50/50, 12.5/87.5, and 0/100, respectively.

As shown in Figure 2, the synthesized Au NPs were characterized by SEM (Figure 2A) and TEM (Figure 2B). They have uniform morphologies and good dispersity. The average diameter of the Au NPs is 30 nm, which results in a distinct SPR peak at ∼520 nm (Figure 2C). The Au nanoarchitecture can be well controlled by adjusting the concentrations of CTAC and CTAB in the synthesis solution (Supplementary Figures S1, S2). The TEM image of representative nanotip from Au nanoarchitecture is shown in Supplementary Figure S2I. The Au NP morphology gradually changed with an increasing CTAB/CTAC ratio, from meatball-like shapes (Figure 2Di, no CTAB) to star-like (Figure 2Dii–vii) shapes, and to spherical-like shapes (Figure 2Dviii, no CTAC). The evolution of the Au NP morphology shows that Br^−^ and Cl^−^ ions play important roles in the growth process. The Au NP evolution is also reflected by the extinction spectra shown in Figure 2E. As the Br^−^/Cl^−^ ratio increases, a new SPR peak appears at approximately 900 nm (Figure 2Ev). This new peak appears because of the lightning-rod effect, in which the nanotip serves as an antenna. Coupling of the core and nanotip increases the effective dipole moment of the tip plasmons ( Maity et al., 2014; Kim and Ha, 2017). Hybridization of the core and tip increases the cross section for excitation of bonding nanostar plasmons compared with that of an individual nanosphere plasmon.

**FIGURE 3:**
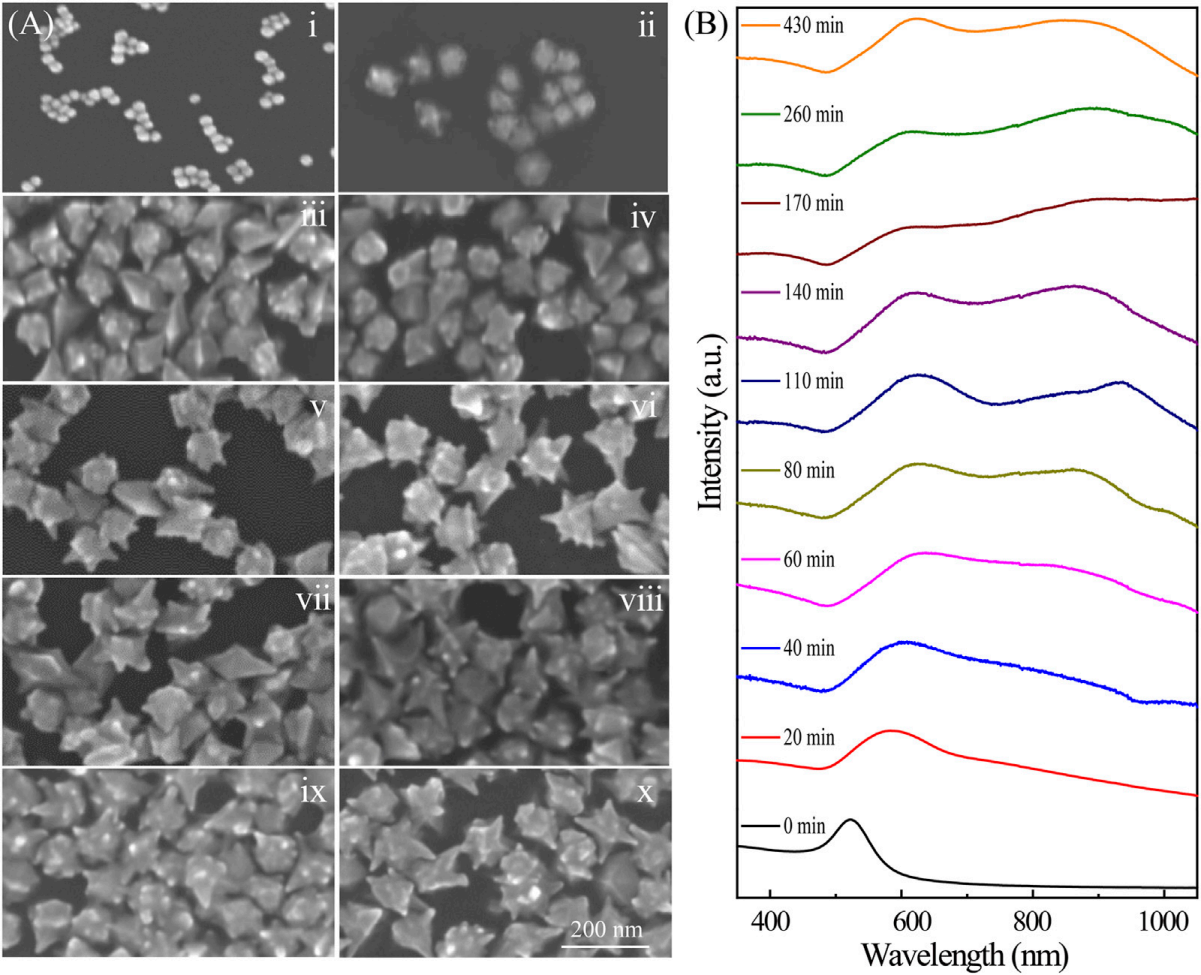
**(A)** SEM images of Au nanoarchitectures synthesized with 0.2 mM AgNO_3_ for different reaction times. i–x correspond to 0, 20, 40, 60, 80, 110, 140, 170, 260, and 430 min, respectively. **(B)** Extinction spectra of Au nanoarchitectures synthesized with 0.2 mM AgNO_3_ for different reaction times.

Further insights into the growth mechanisms of the Au nanoarchitectures were obtained by using SEM to monitor the morphological changes with time, that is, after 0, 20, 40, 60, 80, 110, 140, 170, 260, and 430 min (Figure 3). The Au NPs shown in Figure 2Dv, which were synthesized with CTAC/CTAB concentrations of 62.5/37.5 ⅿⅯ, are discussed in detail. The NP morphology distinctly changed with increasing time, namely, from meatball-like to branched and flower-like shapes. This indicates that the size and morphology of the Au crystals can be changed by controlling the chemical reaction time. The growth process involved in the NP evolution can also be deduced from the extinction spectra shown in Figure 3B. The meatball-like NPs observed at a reaction time of 20 min give a single SPR peak at 571 nm. With increasing time, the SPR peak red-shifts to 630 nm (20–40 min) because of the increased particle size. After particle growth for 60 min, a new SPR peak appears at 630 nm. The corresponding absorption band broadens with increasing time to 160 min. The peak beyond 800 nm in the extinction spectra of the Au nanoarchitectures (20–170 min) gradually appeared and red-shifted because tip growth caused an increase in the effective dipole moment. At reaction times of 170 min and longer, the absorption spectra barely changed, which indicates that no further NP growth occurred. In a typical synthetic procedure, the products usually need to age for at least 3 h to obtain stable Au nanoarchitectures. Here, we describe a new method with a much shorter reaction time, which effectively improves the synthetic efficiency.

**FIGURE 4:**
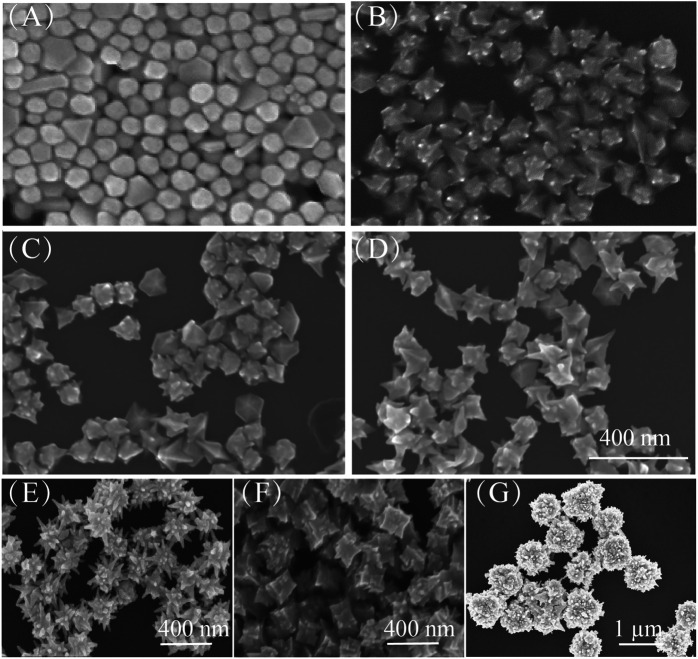
**(A–D)** SEM images of Au nanoarchitectures synthesized with different concentrations of AgNO_3_: 0 mM, 2 mM, 4 mM, and 8 mM. Scale bar is 400 nm. **(E–G)** SEM images of Au nanoarchitectures obtained with different seeds: **(E)** Ag NPs, **(F)** Au nanorods, and **(G)** Au nanoplates.

The growth of Au nanoarchitectures can also be affected by the concentration of Ag ions. Here, Ag ions at various concentrations, that is, 0 mM (Figure 4A), 2 mM (Figure 4B), 4 mM (Figure 4C), and 8 mM (Figure 4D), were used. As shown in Figures 4A–D, when no Ag ions were added, the Au NPs had no sharp points. However, the Au nanoflower tips became larger with increasing Ag ion concentration.

**FIGURE 5:**
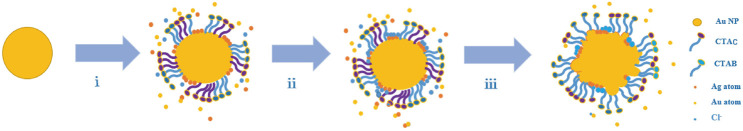
Schematic diagram of growth of proposed Au nanoarchitectures.

Figure 5 shows the growth of Au NPs with tips in detail. When HAuCl_4_ is mixed with CTAC and CTAB, AuCl_4_ ions bond strongly with CTA^+^, and complexes are formed (Khan et al., 2015). On addition of ascorbic acid, the AuCl_4_–CTA complex is partially transformed into AuCl–CTA; Au atoms are steadily released with time, and HAuCl_4_ is gradually reduced. The addition of Ag atoms from AgNO_3_ effectively modifies the Au NP surface (step i). Then Cl^−^ ions are released and adsorbed at Ag sites to form strong Ag–Cl bonds (step ii). Because of the high Ag–Cl binding energy, Au atom migration to, and deposition at, these sites is inhibited. This leads to the formation of abundant nanotips as secondary structures on the Au NP surfaces (step iii) (Fan et al., 2018; Shi et al., 2020). The Au nanoarchitecture can easily be regulated by adjusting the concentration of Cl^−^ ions. This nanotip growth method is suitable for Au NPs of other shape, for example, Ag NPs, Au nanorods, and Au nanoplates ( Figures 4E–G ).

For nanostructures with sharp tips, a strong electric field can be generated in the tip area because of a high aggregation of free charges; the LSPR decay can lead to a rapid and localized thermal effect, which provides the activation energy for surface chemical reactions. We simulated the EM and photothermal enhancement properties of NPs of various shapes. The enhancement effects for NPs with tips were evaluated by performing plasmon-driven catalytic reactions.

Figure 1 shows the simulation results for the EM field and temperature distributions for NPs with various shapes. The shapes correspond to different reaction times for tip growth on Au NPs. Figure 1A shows a schematic diagram of laser-excited Au NPs; the wavelength of the incident light is 633 nm, and the particle is mediated in air. Figure 1B clearly shows that with tip growth, the electric field near the tip area is enhanced at least 30-fold times at an excitation power of 1 mW. This enhancement is five times larger than that for bare nanospheres. Tip growth not only contributes to stronger electric field enhancement but also improves the absorption ability of the NPs (i.e., increases the absorption cross section), which results in highly efficient photothermal conversion. As shown in Figure 1C, for bare NPs, the temperature rise under illumination is only ∼90 K. However, with tip growth, the average NP temperature can increase by ∼186 K, which results in a better platform for photocatalytic reactions.

**FIGURE 6:**
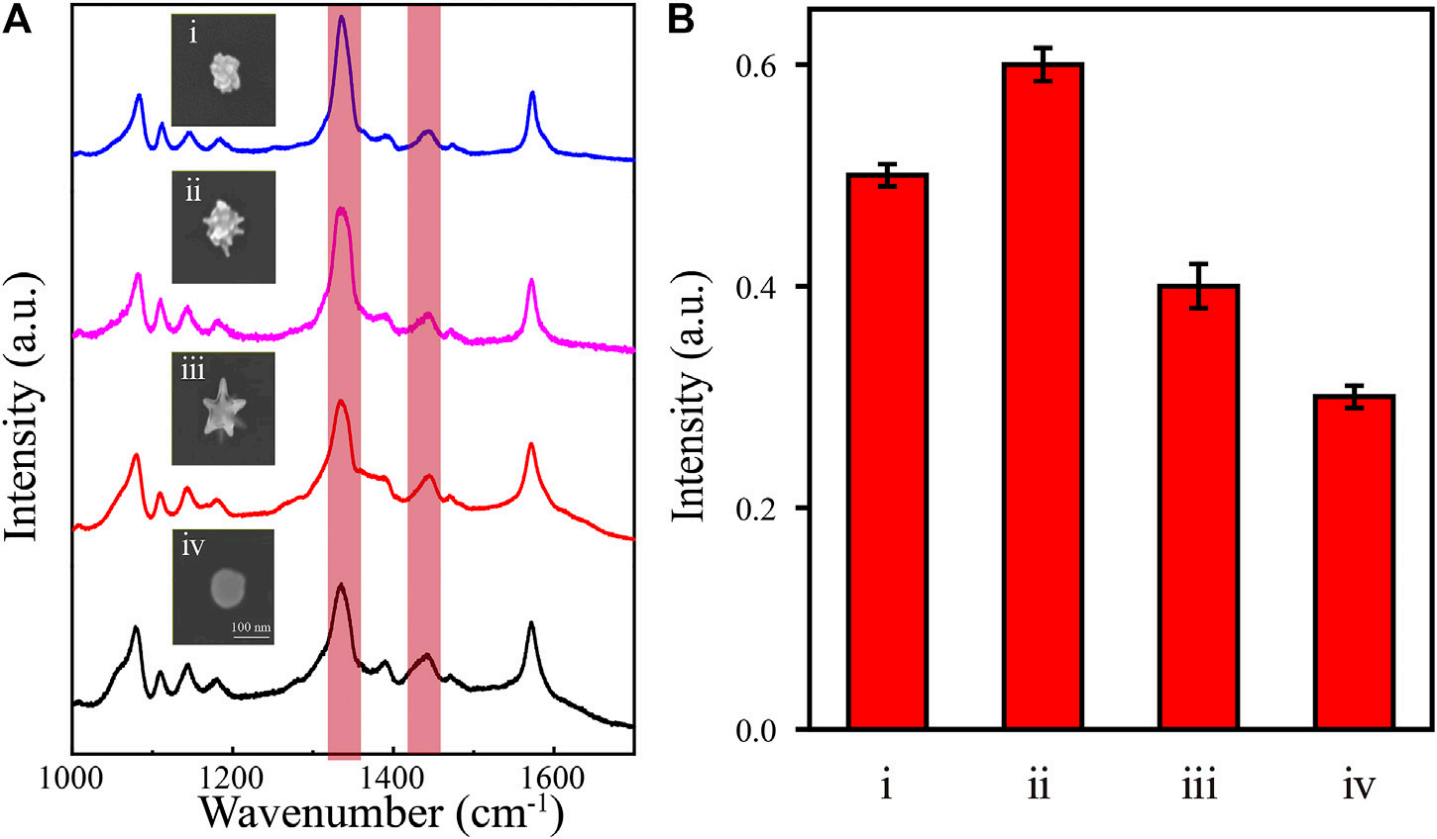
**(A)** SERS spectra of 4-NTP (10^–7^ M) and SEM images of different Au nanoarchitectures. **(B)** The intensity ratio of (1,435 cm^−1^ν_N=N_/1,335 cm^−1^ν_NO2_) for different Au nanoarchitectures; i, ii, iii, and iv correspond to the images in Figure 2Dii, iv, v, and viii, respectively.

In a typical plasmon-catalyzed reaction, 4-NTP adsorbed on plasmonic nanostructures reacts exclusively to give 4,4′-dimercaptoazobenzene (DMAB) (Xie et al., 2013; Zhang et al., 2013; Kim et al., 2014). The plasmon-catalyzed dimerization of 4-NTP to DMAB is characterized by the disappearance of the Raman band at 1,335 cm^−1^ (ν_NO2_) and the appearance of new Raman bands at approximately 1,140 (β_C–H_), 1,387 (ν_NN_ + ν_CC_ + ν_C–N_), and 1,435 (ν_N=N_) cm^−1^ (Xie et al., 2011). The temperature is important in this chemical reaction. We used 4-NTP as a probe molecule to verify the photothermal effect of the synthesized NPs with nanotips. The SERS spectra from 4-NTP with various Au nanoarchitectures were recorded under 633 nm laser excitation at a power of 1 mW (Figure 6A). The results show that a new Raman peak appears at 1,435 cm^−1^, although the peak at 1,335 cm^−1^ is still strong. These features indicate that the catalytic reaction was successfully activated and the 4-NPT molecules were converted to DMAB. As shown in Figure 6B, the photothermal properties of NPs of various shapes were evaluated from the intensities of the peaks at 1,335 and 1,435 cm^−1^ in the SERS spectra. The results show that different Au nanoarchitectures have different photothermal properties. The meatball-like (Figure 3Aii) and star-like (Figure 3Aiv and v) samples are more efficient catalysts than NPs without any tips (Figure 3Aii). This is supported by the EM field and temperature distribution simulations shown in Figure 1. In summary, the photothermal properties of Au nanoarchitectures can be effectively adjusted and controlled by growth of different shapes.

## Conclusion

A new efficient approach for growing nanotips on arbitrary Au nanoarchitectures was developed by simply adjusting the concentrations of CTAC and CTAB in the solution. The surface modification of AuNPs by AgCl leads to the formation of abundant nanotips as secondary structures. This method is not only suitable for growing nanotips on the surfaces of any metal NPs but also avoids surfactant effects. The EM and photothermal field enhancement effects of Au nanoarchitectures with nanotips are better than those achieved with architectures with smooth surfaces. This is because the nanotips lead to stronger light confinement. This was verified by FEM simulations and catalytic experiments. The simulation results show that the electric field enhancement with NPs with large tips is approximately five times that achieved with NPs without tips. In addition, the average temperature of the NPs gradually increases with tip growth because the absorption cross section increases. This provides a good platform for photothermal catalysis. This method provides a new approach to the design of highly efficient steady-state photothermal catalysts. It has potential applications in plasmonic sensors and photothermal treatment in biotechnology.

## Data Availability

The original contributions presented in the study are included in the article/Supplementary Material; further inquiries can be directed to the corresponding authors.
