# Supplementary material for: Binary Surfactant–Mediated Tunable Nanotip Growth on Gold Nanoparticles and Applications in Photothermal Catalysis
Source: Front Chem. 2021 Jul 7;9:699548. doi: 10.3389/fchem.2021.699548 (PMC8294035; doi:10.3389/fchem.2021.699548)
Supplement: Supplementary file 1 [file DataSheet1.docx]

Supplementary Material

**Binary surfactant mediated tunable nanotips growth on** **gold nanoparticles and application in photothermal catalysis**

Xiaohu Mi,^†^ Tingting Zhang,^†^ Baobao Zhang, Min Ji, Bowen Kang, Chao Kang, Zhengkun Fu,^*^ Zhenglong Zhang,^*^ Hairong Zheng

*School of Physics and Information Technology, Shaanxi Normal University, 710119，Xi'an, China*

*Email: zkfu@snnu.edu.cn; zlzhang@snnu.edu.cn

## Supplementary Figures


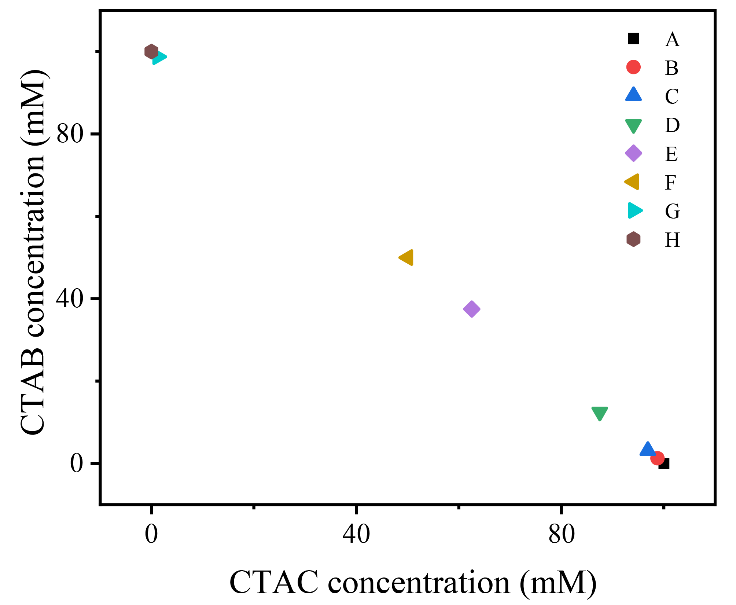


**Figure S1.** Au nanoarchitectures obtained by using different CTAC/CTAB concentrations (mM): (A) 100/0, (B) 98.75/1.25, (C) 96.9/3.1, (D) 87.5/12.5, (E) 62.5/37.5, (F) 50/50, (G) 12.5/87.5, and (H) 0/100.


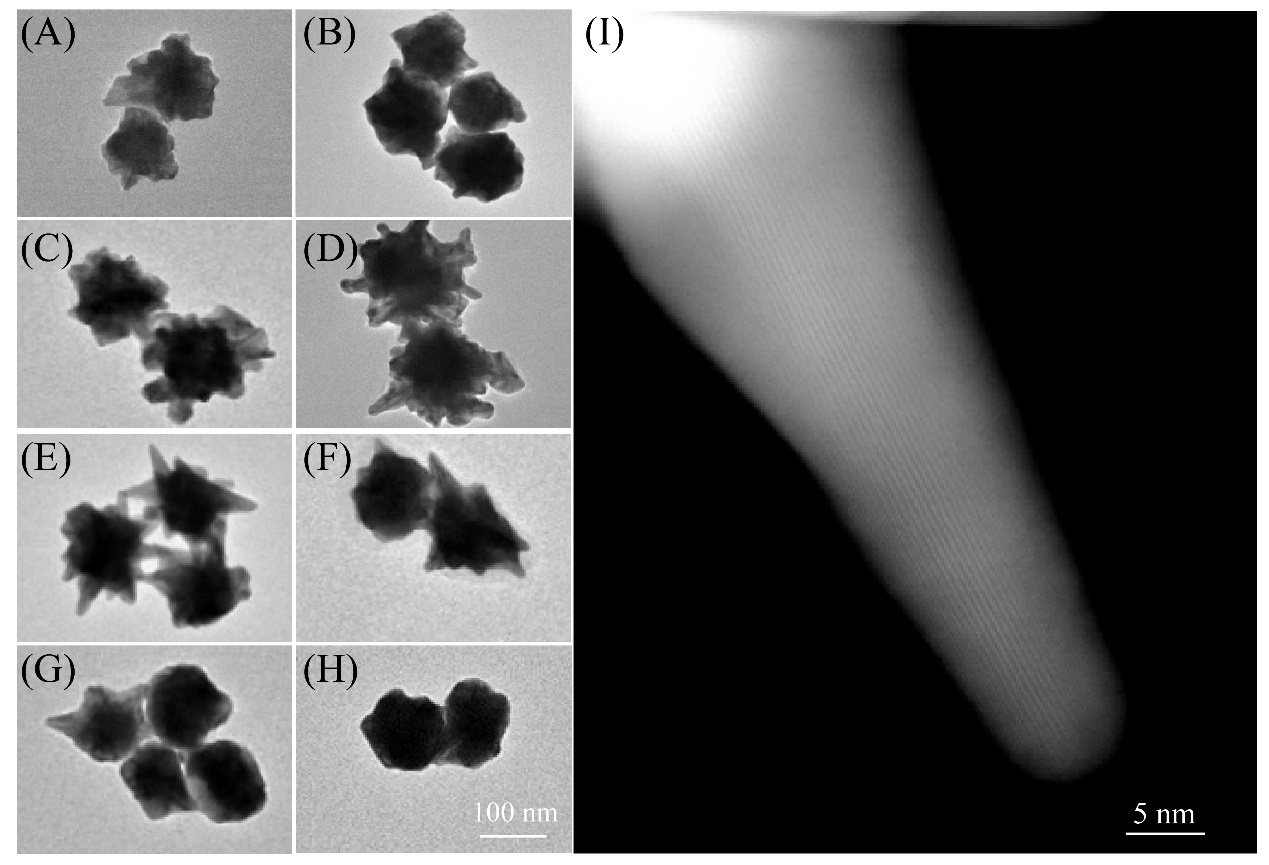


**Figure S2.** TEM images of Au nanoarchitectures obtained with different concentrations of CTAC and CTAB (A–H in Figure S1). (I) The TEM image of nanotip from Au nanoarchitecture.
